# Draft Genome Sequences of *Bordetella holmesii* Strains from Blood (F627) and Nasopharynx (H558)

**DOI:** 10.1128/genomeA.00056-13

**Published:** 2013-03-21

**Authors:** Kathleen M. Tatti, Vladimir N. Loparev, Satishkumar RanganathanGanakammal, Shankar Changayil, Michael Frace, Michael Ryan Weil, Scott Sammons, Duncan MacCannell, Leonard W. Mayer, M. Lucia Tondella

**Affiliations:** a Centers for Disease Control and Prevention, National Center for Immunization and Respiratory Diseases, Division of Bacterial Diseases, Meningitis and Vaccine Preventable Diseases Branch, Atlanta, Georgia, USA; b Centers for Disease Control and Prevention, National Center for Emerging and Zoonotic Infectious Diseases, Division of Scientific Resources, Biotechnology Core Facility Branch, Atlanta, Georgia, USA

## Abstract

*Bordetella holmesii*, a human pathogen, can confound the diagnosis of respiratory illness caused by *Bordetella pertussis*. We present the draft genome sequences of two *B. holmesii* isolates, one from blood, F627, and one from the nasopharynx, H558. Interestingly, important virulence genes that are present in *B. pertussis* are not found in *B. holmesii*.

## GENOME ANNOUNCEMENT

In 1995, Gram-negative, rod-shaped, CDC nonoxidizer group 2 strains first isolated from blood cultures were classified to the genus *Bordetella* and named *Bordetella holmesii* (1). Since *B. holmesii* is found in nasopharyngeal specimens and contains IS*481* (2), *B. holmesii* confounds the diagnosis of *B. pertussis*. Despite the clinical importance of *B. holmesii*, its virulence genes and genome sequence have not been determined. We report the first draft genome sequences of 2 *B. holmesii* strains.

The genomes of strains *B. holmesii* F627 (blood) and H558 (nasopharynx) were generated using a combination of Roche/454 and Illumina technologies with Genome Sequencer (GS) FLX Titanium and Genome Analyzer (GA)IIx instruments, respectively. The raw data and coverage analysis were performed using customized software tools (unpublished data). The coverage depth was referenced to the genome length of *Bordetella avium* (3.7 Mb), as *B. holmesii* sequences were >80% homologous to that of *B. avium*. For strain F627, coverage was 25.8× and 98.2× by 454 and Illumina, respectively, while H558 coverage was 20.4× and 66.5×, respectively, with quality filter of Q20 and a minimum read length filter of 50 bp. *De novo* assembly performed for a hybrid of 454 and Illumina data (CLC Genomics workbench 5.5.1; CLC bio, Aarhus, Denmark) produced the lowest number of contigs with the highest N_50_ value. F627 had 224 contigs with lengths >510 bp, while H558 had 231 contigs with lengths >428 bp. The estimated genome size was 3.8 to 3.9 Mb. To reduce the number of contigs, additional long-read sequencing was performed using the Pacific Biosciences *RS* with libraries containing insert sizes of 1 kb and 5 kb. The PacBio sequences for both F627 and H558 were error corrected with CCS (circular consensus sequence) data and assembled using Celera Assembler (3), resulting in 13 and 30 contigs, respectively.

By comparison of KpnI and NheI digests of F627 and H558, whole-genome optical maps (Opgen, Inc., Gaithersburg, MD) and *in silico*-generated physical maps of contigs confirmed the correctness of the hybrid assembly. The maps permitted the alignment and orientation of 4 contigs for F627 and 9 contigs for H558 and identification of misassemblies, allowing the production of PCR products to cover all remaining gaps in the sequence.

Contigs from hybrid *de novo* assembly were run through custom assembly software that attempts to order contigs and close gaps. The tool ordered and closed the gaps in F627, reducing the contigs to 2, while the tool had low confidence in gap closure for H558, producing 12 contigs.

Draft genome sequences were annotated using the Prokaryotic Genome Annotation Pipeline (PGAAP) at NCBI. The two genomes are structurally very similar. The data demonstrated that adenylate cyclase, filamentous hemagglutinin, and *bvgA* were 70%, 80%, and 85% homologous to those in *B. avium*, respectively. However, fimbriae, pertussis toxin, and pertactin were not found in either strain. Since *B. holmesii* causes similar clinical infectious presentation as *B. pertussis* (4), these results are intriguing, as common virulence factors, importantly the pertussis toxin, are missing in *B. holmesii*.

### Nucleotide sequence accession numbers. 

The draft genome sequence for *B. holmesii* strain F627 has been included in the GenBank Whole-Genome Shotgun (WGS) database under the accession no. AOEW00000000. The version described in this paper is the first version, accession no. AOEW01000000. The draft genome sequence for *B. holmesii* strain H558 has been included in the GenBank Whole-Genome Shotgun (WGS) database under the accession no. AOFR00000000. The version described in this paper is the first version, accession no. AOFR01000000.
